# Punishment a Factor of Inebriety and Insanity

**Published:** 1884-05

**Authors:** T. D. Crothers

**Affiliations:** Supernintedendent Walnut Lodge, Hartford, Conn.


### Selections.

### PUNISHMENT A FACTOR OF INEBRIETY AND INSANITY.

BY T. D. CROTHERS, M. D.

*Superintedendent Walnut Lodge, Hartford, Conn.*

[American Psychological Journal.]

It is asserted by competent authorities that from twenty to fifty per cent. of all insanity can be traced directly to inebriety. Whatever the truth may be, it is evident that inebriety is a factor of great magnitude in the causation of insanity. All conditions which favor the growth of inebriety, both directly and indirectly increase insanity. The one is tributary to the other. Large armies of inebriates are followed by field hospitals for the insane, and jails, almshouses, and asylums for all forms of degeneration and disease that grow out of them. All progress of humanity and civilization turns on the recognition and prevention of those factors which destroy the physical and mental health of the individual and nation. No study of means for the prevention of insanity can ignore inebriety as a factor, with its positive and wide reaching influences. These are truisms. To day one of the great problems that demands recognition and solution is this; there are over three hundred thousand inebriates in this country, unknown, neglected, diseased, and steadily growing worse. An army from which not only insanity is recruited, but criminality, pauperism, and the vast hordes of the defective classes, which are an increasing menace to law and order, industry and all healthy progress. It is a problem that enters into our homes and firesides, and touches all our individual and social interests. This fact is still unrecognized, and the effort to solve the problem as a moral evil, intensifies and magnifies the disorder which it seeks to remove.

In the last century insanity was increased and made more incurable by the application of means and remedies that failed to comprehend the true condition of the insane. New York State found that pauperism was fostered and increased in the almshouses, where they sought to check and remove it Prisons and jails have been literal training schools for crime, and their inmates made more dangerous by their confinement. To-day the legal treatment for inebriety, by fines and imprisonment, makes the victim more incurable, and increases his physical and mental degeneration.

Thus history repeats itself, and the prosecution of the neurotic and insane of the last century, as "possessed of the devil." is repeated in the prosecution of the inebriate of to-day, as being willful and wicked, in voluntarily choosing a course of ruin and destruction. Thus public sentiment, by favoring the punishment of inebriates not only increases the malady, but multiplies the tendency to insanity. The object of the law is reversed; instead of checking inebriety it increases it.

HISTORICALLY, this is clear, from the fact that the criminal code concerning inebriety is just where it was twenty-three hundred years ago, when Epicurus taught that inebriety could only be prevented by punishment and physical suffering. Through all the ages of progress, the civilization of punishment relating to inebriates has stood still. To-day, the judge on the bench, and the teacher from the pulpit, are repeating the same theories, denying all progress of science and increased knowledge of the brain and its diseases. It is the same superstition which, from the earliest times, has ever ascribed a spiritual origin to all phenomena that were not understood.

As humanity moves on, all theories of the vice and wickedness of inebriety should be settled from the facts in the light of the day, and not from the teachings of the early ages. It is from this misconception of the nature and character of the disease that the legal treatment of intemperance becomes an active cause in increasing and continuing it. It is assumed that every inebriate can abstain at will, and is always a free agent in the use of alcohol. That his failure to abstain is the result of a bad, vicious disposition, and reckless disregard of duty and of the rights of others. No matter what his real condition may be, the legal remedy is the same. He may be an epileptic, drinking to excess after the paroxysm; or a neurotic, with the entailment of past generations of disease, and the drinking a mere symptom of such degeneration; or an imbecile, with defective brain organization; or a victim of brain and nerve exhaustion, who turns to alcohol for relief. It matters not what his unrecognized physical state, in court he is always and ever a degraded sinner, who can only be reached by punishment.

The principle of law in relation to inebriates is false, and opposed by all teachings of nature and science. A broken bone is never healed, or the eyes of the blind restored, except by the application of exact means, and with a full recognition of the nature and character of the injury. No physical appeal, through fear, shame and suffering, can ever build up a weakened brain or nervous system. Hence the legal means to reach and check inebriety must intensify it, and always fail, because they are aimed at conditions that do not exist.

THE STATISTICAL RESULTS OF THE LEGAL TREATMENT OF INEBRIATES furnish the most convincing evidence of the disastrous consequences which follow from such means.

At the International Prison Congress, in 1871, it was asserted that not one in a thousand persons committed to jail for inebriety ever recovered. Several witnesses, of large experience in jails and lower courts of England, testified before the Committee of the House of Lords, that they had never heard of a case of restoration of inebriates from punishment by fine and imprisonment, but that they invariably became repeaters and chronic incurables.

This experience is confirmed by judges and prison authorities all over the country. Inquiries addressed to ten different penitentiaries who receive prisoners of this class, from the largest cities, brought out nine answers, emphatically declaring that no instances of the recovery of inebriates who were committed for this cause were known. But that in the vast majority of cases the first sentence for this cause was speedily followed by others, and unless the victim died or moved away, his return was a matter of certainty, year after year.

This fact is supported by startling statistics, from which we select a few examples: In 1879 Massachusetts punished, by imprisonment and fine, over seventeen thousand inebriates, more than sixteen thousand of whom had been in prison before for the same cause. In New York fifty-six thousand inebriates came under legal notice in 1852, and were punished by fine and imprisonment. Less than one thousand of this number were committed or punished for the first time; all the others were repeaters, and had been sentenced before for the same cause. The same year, in Connecticut, thirtysix hundred inebriates were sent to jail. All but about three hundred of this number had been in jail before, or been fined, for the same offense.

These statistics can be multiplied almost indefinitely, but are sufficient to show that such punishment does not check inebriety or cure the inebriate. But, on the contrary, it builds up a class of incurables, from which the most dangerous elements of society spring. An examination of the records of any lower court in the large cities will show that two-thirds of all the business is the commitment of chronic inebriates over and over again, year after year. A visit to any jail in the neighborhood of large cities will indicate a large per cent. of all its inmates who are inebriates that have been sentenced before for the same cause. Judges and prison anthorities are powerless; they must see passing under their control and observation the development and mobilizing of an army of incurables that they cannot check. The unfortunate victim who for the first time serves out a sentence for inebriety must come in contact and be ranked with the low felon, and the worst elements of his nature aroused and strengthened Both judge and jailor realize that the inebriate has crossed the frontier into the broad highway of disease, crime, insanity and death, but in obedience to a public sentiment that constitutes them the agents to accelerate his degeneracy, and organize him into the great army of the defectives.

HOW THE PRISON TREATMENT OF THE INEBRIATE MAKES HIM INCURABLE is apparent from the most cursory examination.

Under all circumstances he is enfeebled and diseased. His brain is starved from defective nutrition; alcohol has perverted healthy nerve activities, and his normal vigor and force are lowered. In a large portion of cases he has inherited diseased tendencies; in other cases he has suffered from overwork, or under-work, from exhaustion, and general neglect of all healthy living, or he is a victim of neurotic disease, insidious are pronounced, and while alcohol intensifies and develops these neurotic states, its use is more often a symptom of active degeneration, and an exciting cause of conditions that come from many and widely diverging events.

Penal treatment removes the victim from alcohol only, neglecting every other condition of ill health and disease. Prominent among these neglected means for restoration is unsuitable diet. No prison or jail furnishes proper food to build up healthy nerve and brain tissue. It is always wanting in quality, variety and form. It is furnished to persons who are supposed to be vigorous and healthy, and is not intended to build up a diseased body and brain. Hence the inmates are badly nourished and their nervous systems starved in many ways. Ventilation and sunlight are always defective. The long, narrow cells, under the best conditions, teem with poisonous odors, and a healthy circulation of pure air has never been an accomplished fact. Exercise, in the best circumstances, is also wanting in variety and conditions. All these facts, and more, are most strikingly confirmed by the pale, bloodless faces and feeble pulse beats of nearly all prisoners. Anæmia, malnutrition, steady blood poisoning, hurry on the victim to consumption, the scourge of all prisons.

Add to all these pronounced physical states, the mental effects of close confinement in gloomy cells, on a level with all kinds of criminals, and all individuality merged into a stream of outlaws from all that is good and true in the world, and nothing seems wanting to make degeneration complete. All the central forces of faith and pride merge into despair and recklessness, and the patient is psychologically, as well as physically diseased. He has crossed the border land into a new realm of obloquy and degradation, from which he rarely ever comes back. Thus the State, in the punishment of inebriety, with a cruelty that is thoroughly scientific in its certainty and precision, puts the poor neurotic inebriate in bad ventilated places, with unsuitable food, in depressing surroundings, associated with the lowest and most degraded classes, permanently crippling him for all the future. The poet's inscription, "he who enters here leaves hope behind," has a literal reality to all inebriates who are sentenced to prison for the first time.

Such is the result of the present treatment of inebriety, based as it is on the theory of two thousand years ago, and which finds its defenders among the moralists and pseudo-reformers of the nineteenth century.

The time has come to demand that the inebriate be regarded from the standpoint of science; that other and more exact means be applied, which, if it cannot cure the victim, will not intensify his malady, and make him a centre of infection to others.

THE TRUE REMEDY FOR THIS

will be found in work-house hospitals for the inebriate. Buildings erected on large farms, removed from great centres, conducted on a emi-military plan, where occupation, restraint, education, and physical and moral treatment may be carried out with skill and exactness. Where every appliance of science and art can be applied which shall build up the weakened body and brain Where the curables can be restored to the ranks of producers, and the incurables can be housed and made self supporting. Where all the vast tides of evil that spring from inebriety can be reduced to a minimum. Where the epileptic, paralytic, neurotic, congenital and worn-out inebriate can find rest and help and possible restoration from the inevitable insanity, criminality, pauperism, and violent death before him Such institutions might be built from the license fund, and independent of the taxpayer, supported from the income and labor of patients. Each inmate should be self-supporting, from well directed congenial labor, and all means should be applied to develop the best physical health of the inmates. The possibility of the success of such hospitals is already assured in many penal institutions. Take the Albany penitentiary as an example. Here, from less than a thousand inmates of the lowest classes, serving short sentences, an income of thirty and forty thousand dollars is made yearly, above expenses.

Other institutions prove that inebriates sentenced for long terms, and not treated as felons, but as members of a well disciplined household, can be trained into great usefulness, and many be permanently restored.

It also indicates that in this way society can be relieved of the burdens of the incurables, and thus the highest achievement of sanitary science, the prevention of disease and its entailments, can be attained in a large measure. The incurable insane are now housed in many States, to the great advantage of society, and their broken-down energies turned into useful channels. The incurable inebriate is a much more hopeful and promising case. In the proper surroundings, under careful management, he can be made independent and self-supporting more positively than any other.

He is a pronounced neurotic and cannot be treated with criminals or insane, but must have special surroundings and special care. From a

### CLINICAL POINT OF VIEW

the facts are of startling significance. Go into any court and watch those who are daily sentenced for inebriety. In almost every case you will see ill-shapen heads and defective organization, or other unmistakable signs of unsound and diseased brains. The face bears the marks of ungoverned impulses, of neglect, suffering and want, and the whole man has the stamp of transmitted or acquired degeneration.

They are of unsound oodies and unsound minds, diseased outgrowths of both the past and present. These facts stand out so prominently that no expert is needed to determine them. If we inquire into the history of any one of these cases, we shall find physical neglect and unhealthy conditions of life for years; we shall find perverted brain activities, and unsound thoughts and deeds; we shall find that the healthy governing centres of life are becoming more and more disorganized; and, in brief, the whole man has been diverging further and further from the main line of health.

With these most positive indications of diseased bodies and minds, they are sentenced for days, months and years, to prison, and kept in the most unsanitary conditions of ventilation, sunlight, food, exercise, and physical advantages, and are then expected to go out strong, vigorous and temperate, and of sound mind ever after. Such a result is impossible. But just such conditions are the fertile soils and breeding grounds for insanity, criminality, and the lowest forms of pauperism.

A noted lawyer, in a recent address on prison sanitation, remarks as follows: "The more extended study and investigation one gives to this subject, the more clearly the fact will appear, that disease lies at the basis, and is the genesis of by far the largest percentage of crime, and that these poor and friendless outcasts, who are the pariahs of society, and against whom almost every man's hand is raised, should be treated by the physician, and not turned over to the cruel and heartless treatment of the jailor, shut up in prison walls, where every condition of health is violated, and a cure made impossible. Is it a wonder that these poor creatures, when released, turned out again to prey upon society, should feel that their hands should be raised against every man; that they should become the wild Ishmaels of society, carrying into it the blackness and desolation of their own sad lives?" This is true of the vast armies of inebriates, from whom are made insane criminals, demanding special and separate care. To the producer and tax-payer the penal treatment of this class is most disastrous. Instead of lessening the burdens and perils of industry, it increases them.

Society, and even civilization, is periled by the increasing number of diseased and defective members.

While we may not be able to realize and apply all the means to check and prevent their growth, we can avoid all misdirected remedies which intensify and increase the number of victims. To this end a reform is demanded in the penal treatment of the defective classes. My purpose has been merely to outline in this paper the facts which are daily becoming more prominent, and to call attention to them, and the remedy which must be applied sooner or later. A summary of them may be stated as follows:

1. Inebriety is well recognized as an active cause of insanity. Its study is essential to successfully apply the means of prevention and cure of insanity.

2. The penal treatment of inebriety utterly fails to check or cure it. This is apparent from the fact that it is based on a false theory of the nature and character of the disease, and applies the same remedy for widely differing physical states.

3. Statistics show that penal treatment makes the inebriate worse, precipitating him into chronic conditions, from which recovery is very rare. This is the natural result of bad prison sanitation and neglect of all healthy recuperative physical forces, together with the demoralization resulting from contact with felons.

4. The remedy is in special work in hospitals, conducted on a military plan, where the inmates may be self supporting, and have all the best medicinal, educational and hygienic influences known to science, to rouse up and strengthen them for the future.

5. Lastly, the medical treatment of inebriety is a question of sanitary importance of the widest interest; a question of social science and civilization that cannot be ignored; also a question of humanity and economy, concerning the army of the diseased and defectives, that must be solved from a scientific study of the facts.

